# Outcomes Associated with First-Line anti-PD-1/ PD-L1 agents vs. Sunitinib in Patients with Sarcomatoid Renal Cell Carcinoma: A Systematic Review and Meta-Analysis

**DOI:** 10.3390/cancers12020408

**Published:** 2020-02-10

**Authors:** Carlo Buonerba, Pasquale Dolce, Simona Iaccarino, Luca Scafuri, Antonio Verde, Ferdinando Costabile, Martina Pagliuca, Rocco Morra, Vittorio Riccio, Dario Ribera, Pietro De Placido, Valeria Romeo, Felice Crocetto, Nicola Longo, Ciro Imbimbo, Sabino De Placido, Giuseppe Di Lorenzo

**Affiliations:** 1 Regional Reference Center for Rare Tumors, Department of Oncology and Hematology, AOU Federico II of Naples, 80131 Naples, Italy; 2 National Reference Center for Environmental Health, Zoo-prophylactic Institute of Southern Italy, 80055 Portici, Italy; 3 Department of Public Health, Federico II University of Naples, 80131 Naples, Italy; 4 Department of Clinical Medicine and Surgery, University Federico II of Naples, 80131 Naples, Italy; 5 Department of Advanced Biomedical Sciences, University of Naples “Federico II”, 80131 Naples, Italy; 6 Department of Neurosciences, Human Reproduction and Odontostomatology, University of Naples Federico II, Naples 80131, Italy; 7 Department of Oncology, Hospital “Andrea Tortora”, ASL Salerno, 84016 Pagani, Italy; 8 Department of Medicine and Health Sciences ‘Vincenzo Tiberio’, University of Molise, 86100 Campobasso, Italy

**Keywords:** renal cell carcinoma, sarcomatoid, immune checkpoint inhibitors, PD-1, PD-L1

## Abstract

Immunotherapy based on anti PD-1/PD-L1 inhibitors has proven to be more effective than sunitinib in the first-line setting of advanced renal cell carcinoma (RCC). RCC patients with sarcomatoid histology (sRCC) have a poor prognosis and limited therapeutic options. We performed a systematic review and a meta-analysis of randomized-controlled trials (RCTs) of first-line anti PD-1/PDL-1 agents vs. sunitinib, presenting efficacy data in the sub-group of sRCC patients. The systematic research was conducted on Google Scholar, Cochrane Library, PubMed and Embase and updated until 31th January, 2020. Abstracts from ESMO and ASCO (2010–2019) were also reviewed. Full texts and abstracts reporting about RCTs testing first-line anti-PD-1/ PD-L1 agents vs. sunitinib in RCC were included if sRCC sub-group analyses of either PFS (progression-free survival), OS (overall survival) or radiological response rate were available. Pooled data from 3814 RCC patients in the ITT (intention-to-treat) population and from 512 sRCC patients were included in the quantitative synthesis. In the sRCC sub-group vs. the ITT population, pooled estimates of the PFS-HRs were 0.57 (95%: 0.45–0.74) vs. 0.79 (95% CI: 0.70–0.89), respectively, with a statistically meaningful interaction favoring the sRCC sub-group (pooled ratio of the PFS-HRs = 0.64; 95% CI: 0.50–0.82; *p* < 0.001). Pooled estimates of the difference in CR-R (complete response-rate) achieved with anti-PD-1/PDL-1 agents vs. sunitinib were + 0.10 (95% CI: 0.04–0.16) vs. + 0.04 (95% CI: 0.00–0.07) in the sRCC vs. the non-sRCC sub groups, with a statistically meaningful difference of + 0.06 (95% CI: 0.02–0.10; *p* = 0.007) favoring the sRCC sub-group. Sarcomatoid histology may be associated with improved efficacy of anti PD-1/PDL-1 agents vs. sunitinib in terms of PFS and CR-R.

## 1. Introduction

Renal cell carcinoma (RCC) represents the ninth and 14th most commonly diagnosed cancers in men and women, respectively, with an estimated >400,000 new RCC cases reported in 2018 [1] and a cumulative life-time mortality risk varying in range of 0.1–0.7% worldwide [2]. Western Europe and North America present the highest age-standardized incidence rates [2]. Metastatic RCC is virtually incurable, although median survival among risk groups has approximately doubled with the advent of targeted therapy [3]. The use of anti PD-1 (programmed death 1) / PD-L1 (programmed death - ligand 1) agents in combination with anti-VEGF (vascular endothelial growth factor) or anti CTLA-4((Cytotoxic T-Lymphocyte Antigen 4) agents [4,5] has led to further improvements in the life expectancy of patients with metastatic RCC. Importantly, while anti-VEGF therapy is effective regardless of the prognostic risk-group, ipilimumab + nivolumab vs. sunitinib was effective in intermediate- and poor-risk patients but not in patients at good prognosis, which has made it finally compulsory for risk class to be incorporated in the therapeutic algorithm.

Adverse histologic features, including necrosis, lymphovascular invasion, high nuclear grade, rhabdoid as well as sarcomatoid histology have a remarkable prognostic value, even in patients with small renal masses [6]. Sarcomatoid histology, which is characterized by the loss of typical epithelial features (“dedifferentiation”), is reported in approximately 5–10% of cases, most frequently in clear-cell RCC [7]. In metastatic RCC patients, sarcomatoid histology was associated with a poor median disease-specific survival of 9 months [6], with chemotherapy and targeted therapy showing disappointing efficacy in these patients [8]. Anti PD-1/PDL-1 agents may be more effective in patients with sarcomatoid histology, possibly because of a higher PD-L1 expression [9] and a higher mutational burden [10].

This systematic review and meta-analysis aims to provide a pooled analysis of the available efficacy data of anti PD1/PDL-1 -based therapy vs. sunitinib in patients with sarcomatoid RCC (sRCC) With the limitations imposed by data availability, exploratory analyses were performed to assess differences in efficacy of anti PD-1/PD-L1-based therapy vs. sunitinib between sarcomatoid and unselected/non-sarcomatoid RCC patients, as well as establishing the potentially optimal anti PD-1/PD-L1-based regimen against sRCC.

## 2. Methods

The search for relevant articles was performed by querying PubMed/MEDLINE, EMBASE, Google Scholar, Cochrane Library according to the PRISMA guidelines. The search terms included the following keywords: “sarcomatoid”, “renal cell carcinoma”, “kidney cancer”, “immunotherapy”, “PD-1”, “PD-L1”, “avelumab”, “atezolizumab”, “pembrolizumab”, “nivolumab”.

Articles published since inception until January, 31st 2020 were evaluated for inclusion in the systematic review. Abstracts and presentations from ASCO (American Society of Clinical Oncology) and ESMO (European Society of Medical Oncology) between 2010 and 2019 were also reviewed.

Ten investigators (SL, IS, VA, CF, PM, RD, MR, RV, CF, DPP) worked in two separate groups that independently screened potentially relevant abstracts and retrieved the full texts of articles original data obtained with anti PD-1/PD-L1 agents in RCC.

Full papers and abstracts/presentations reporting original data obtained in RCC patients treated with anti PD-1/PD-L1 agents were assessed for inclusion in the systematic review. Articles reporting data about randomized-controlled clinical trials (RCTs) testing an anti PD-1/anti PD-L1 agent vs. sunitinib in patients with advanced RCC treated in the first-line setting were included in this meta-analysis if sub-group analysis of either PFS, OS or radiological response rate were available in sRCC patients, either in the same publication or in a separate article or abstract. Abstracts were only included if providing original data useful for the purposes of this meta-analysis that were unavailable as a full paper.

Articles cited in the full papers assessed were also evaluated for inclusion in this meta-analysis. In case of duplicate publications, the most updated data were considered. Disagreements about studies were discussed among all investigators and resolved by CB. From each included study, the following data were extracted: name of study, first author and year of publication, study design and blinding, study phase, number of patients, median OS and PFS in the overall population and in the sarcomatoid sub-group, hazard ratio for PFS and/or OS in the overall population and in the sarcomatoid subgroup, partial and complete radiological response rates in the overall population and in the sarcomatoid subgroup. The quality of the included RCTs was assessed using the Jadad scale [11].

## 3. Data Analysis

The primary objective of the meta-analysis was to obtain pooled estimates of the partial response rates (PR-Rs), the complete response rates (CR-Rs), the hazard ratios for progression or death (PFS-HRs) and the hazard ratios for death (OS-HRs), reported in the sub-group of patients with sarcomatoid histology enrolled in RCTs of anti-PD-1 based therapy vs. sunitinib as first-line treatment of advanced RCC. The secondary objectives were to estimate differences in pooled PFS-HRs and OS-HRs between the sub-group of sRCC patients vs. the ITT population as well as to measure differences in the absolute improvements in PR-Rs and in the CR-Rs associated with use of anti-PD-1/PD-L1 based therapy vs. sunitinib (ΔPR-R and ΔCR-R) in the non-sRCC vs. in the sRCC sub-groups.

For PR-R and CR-R, data were presented as percentage and proportion difference with the 95% CIs, while for PFS-HR and OS-HR data were presented as HRs with the 95% Cis. We evaluated heterogeneity among studies using the χ^2^ Q test and I^2^ statistics. For the Q test, significant heterogeneity was declared if *p* < 0.05, while I^2^ values >50% were considered to indicate evident heterogeneity.

Publication bias was evaluated by visual asymmetry on funnel plots of PFS-HR against standard errors, in sarcomatoid sub-group and ITT population. Moreover, a regression test for funnel plot asymmetry was performed to verify whether the association between effect sizes and the related standard errors was statistically significant.

To explore differences in PFS-HR and OS-HR in patients with sarcomatoid histology vs. the ITT population as well as to compare proportion differences for PR-Rs and CR-Rs in patients with vs. patients without sarcomatoid histology, an interaction test was performed following the approach reported by Fisher et al. [12]. The pooled estimate of the HRs for progression or death (pooled PFS-HRs) and for death (pooled OS-HRs) in the sarcomatoid and in the ITT population and the pooled combination of the trial-specific interaction ratios of PFS- and OS-HR between the sarcomatoid vs. the ITT populations (pooled ratios of the PFS-HRs and OS-HRs) were obtained using random-effects models and reported with their corresponding 95% C.I. The pooled estimate of PR-Rs and CR-Rs with anti PD-1/PDL-1 agents vs. sunitinib (pooled PR-Rs and CR-Rs), as well as of the trial-specific interaction proportion differences in PR-Rs and CR-Rs with anti PD-1/PDL-1 agents vs. sunitinib between the sRCC vs. non sRCC sub-groups (pooled differences of ΔPR-R and ΔCR-R) were obtained using random-effects models and reported with their corresponding 95% C.I [12].

The results were also graphically displayed using forest plots where sRCC sub-group were compared to the entire ITT population (PFS- and OS-HR) or the non-sRCC subgroup (PR-Rs and CR-Rs). The pooled estimates were recalculated after excluding each single study according to a Leave-One-Out-analysis in order to evaluate the robustness of results and to show whether a single study influenced the overall estimate of the meta-analysis. The Leave-One-Out-analysis was applied for evaluation of the pooled ratio of the HRs for death and for progression/death and of the complete response and partial response rates.

Finally, a network meta-analysis was performed to compare the efficacy of each therapy with all the other therapies. However, due to the insufficient number of studies for each treatment (i.e., one trial for treatment, expect for one treatment, for which two studies were considered), we limited the analysis providing only a ranking of treatments from greatest to lowest efficacy, and results must be interpreted with caution. On the basis of a frequentist treatment ranking method, we computed the P-scores, which measure the extent of certainty that one treatment is better than another treatment, averaged over all competing treatments [13].

The statistical software R, version 3.6.0 (13) was used for all statistical analyses. Meta-analysis was performed using metafor package, version 2.1–0. The *p*-value < 0.05 was considered as statistically meaningful. Network meta-analysis was performed using the netmeta R package [14].

## 4. Results

### 4.1. Eligible Articles

**Figure 1:**
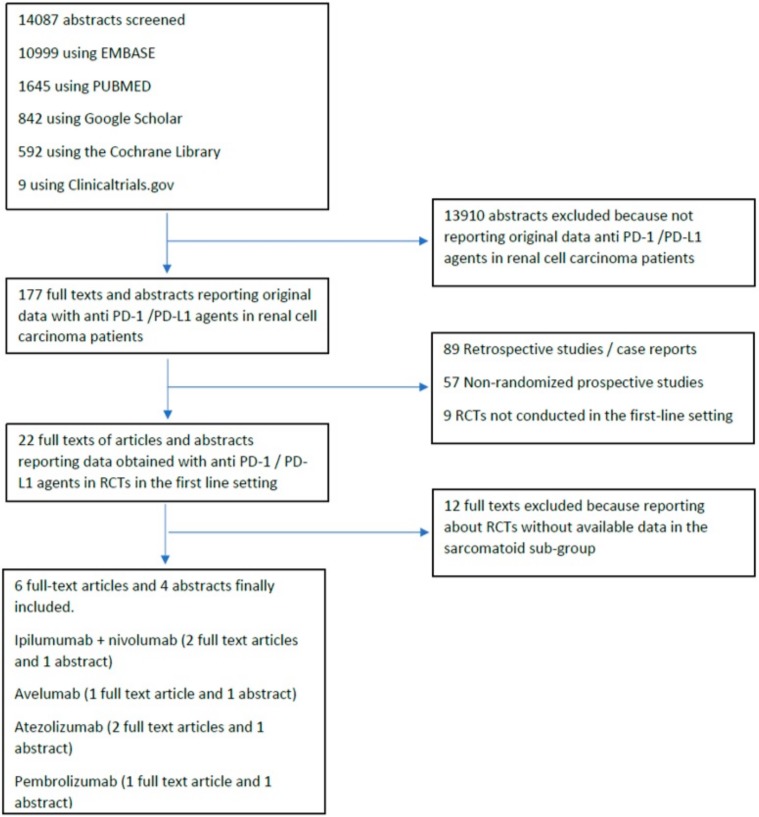
Flow-diagram of the systematic review.

Our database search retrieved 14087 abstracts. Of the 177 full-texts of clinical studies involving the use of anti PD-1/PD-L1 agent in RCC that were evaluated, 6 full text articles [15,16,17,18,19,20] reporting data from RCTs were finally included in the quantitative meta-analysis. Furthermore, four abstracts [21,22,23,24] reporting sub-group analysis in sRCC were also included in the quantitative meta-analysis. The flow chart of the systematic review is reported in Figure 1.

Overall, the six full texts and four abstracts included reported original data from five different RCTs of anti PD-1/PDL-1 based therapy vs. sunitinib obtained in the ITT population and sRCC sub-group.

### 4.2. Quantitative Synthesis

Five different open-label RCTs of anti PD-1/PD-L1 therapy vs. sunitinib were included. Four RCTs were two-arm phase III trials [15,16,17,18,19], while one was a three-arm phase II trial [20]. All five RCTs included had a Jadad score of 3. Relevant findings of the trials included are reported in Table 1. Pooled data from a total of 3814 RCC patients in the ITT population and from a total of 512 patients in the sarcomatoid RCC sub-group were included in the quantitative synthesis.

**Figure 2:**
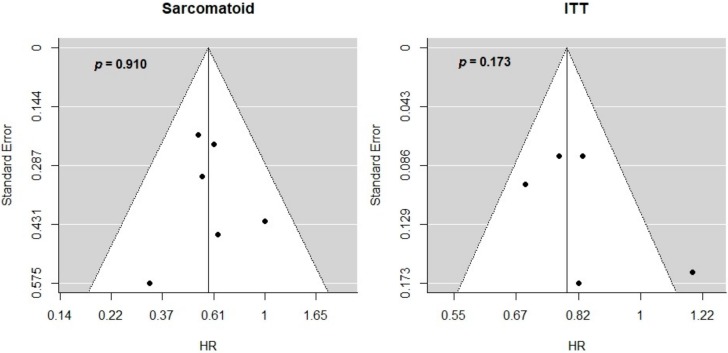
Interactions between HR for progression or death and sarcomatoid histology. Funnel plots of PFS-HR for sarcomatoid sub-group and ITT population. Publication bias was evaluated by visual asymmetry and *p*-values are obtained by regression tests for funnel plot asymmetry. The *y*-axis reports standard error while the *x*-axis reports the effect sizes, that is, HR for PFS in sarcomatoid sub-group (left-hand size) and ITT (right-hand side).

Considering PFS-HR, the included studies did not show an evident reporting bias for both sarcomatoid sub-group and ITT population (Figure 2). Moreover, the regression test did not suggest a significant asymmetry in the respective funnel plots (*p* = 0.910 for sarcomatoid and *p* = 0.173 for ITT population).

**Figure 3:**
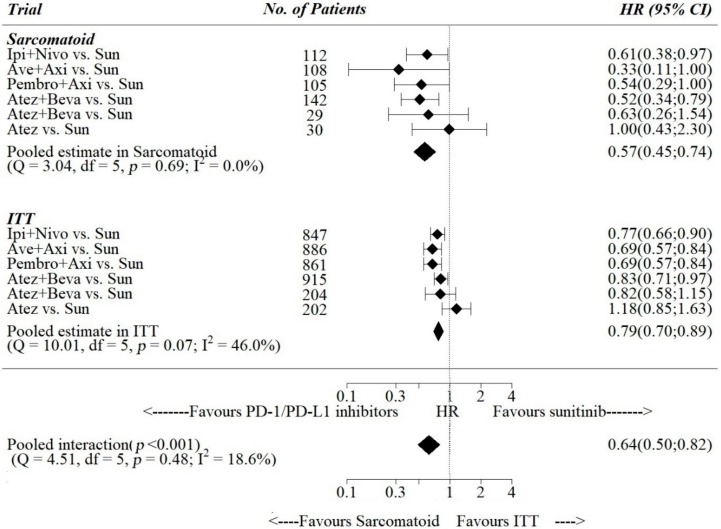
Interactions between HR for progression or death and sarcomatoid histology. Ipi = Ipilimumab; Nivo = Nivolumab; Sun=Sunitinib; Ave=Avelumab; Axi = Axitinib; Atez = Atezolizumab; Beva = bevacizumab; Pembro = pembrolizumab; Sun = sunitinib.

**Figure 4:**
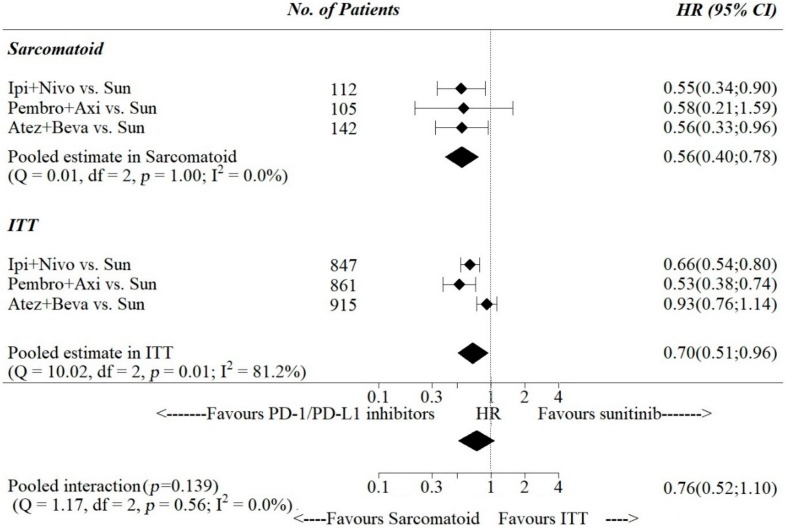
Interactions between HR for death and sarcomatoid histology.

Pooled estimates of PFS -HR were 0.79, 95% [0.70; 0.89] in the ITT population and 0.57, 95% [0.45; 0.74] in the sarcomatoid-only subgroup (Figure 3), while for OS-HR pooled estimates were 0.70, 95% [0.51; 1.10] in the ITT population and 0.56, 95% [0.40; 0.78] in the sarcomatoid-only subgroup (Figure 4). The pooled ratio of PFS-HRs and OS-HRs in the sarcomatoid vs. the ITT population were 0.64, 95% [0.50; 0.82] and 0.76, 95% [0.52; 1.10], respectively. Pooled interaction was statistically significant for PFS-HRs (*p*-value for interaction < 0.001), while it was not statistically significant for OS-HRs (*p*-value for interaction = 0.139).

**Figure 5:**
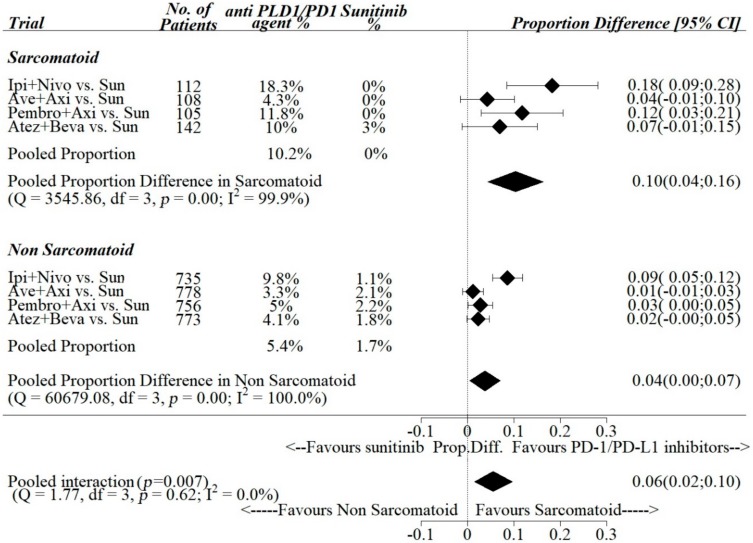
Interactions between complete response rate and sarcomatoid histology.

**Figure 6:**
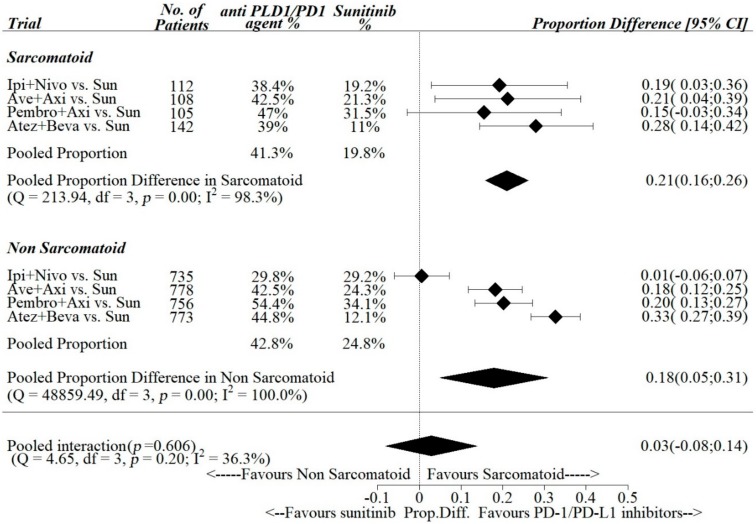
Interactions between partial response rate and sarcomatoid histology.

Pooled estimates of ΔCR-R between anti- PD-1/PDL-1 agents vs. sunitinib among the trials were 0.10, 95% [0.04; 0.16] in the sarcomatoid subgroup and 0.04, 95% [0.00; 0.07] in the non-sarcomatoid sub group (Figure 5), while pooled estimates of ΔPR-R were 0.21, 95% [0.16; 0.26] in the sarcomatoid subgroup and 0.18, 95% [0.05; 0.31] in the non-sarcomatoid sub group (Figure 6).

The pooled differences in the sarcomatoid vs. non-sarcomatoid sub groups of ΔCR-R and ΔPR-R between anti PD-1/PDL-1 vs. sunitinib were 0.06 (95% CI: 0.02–0.10) and 0.03 (95% CI: 0.08–0.14), respectively. Pooled interaction was statistically significant for complete responses (*p*-value for interaction = 0.007), while it was statistically not significant for partial responses (*p*-value for interaction = 0.606).

**Figure 7:**
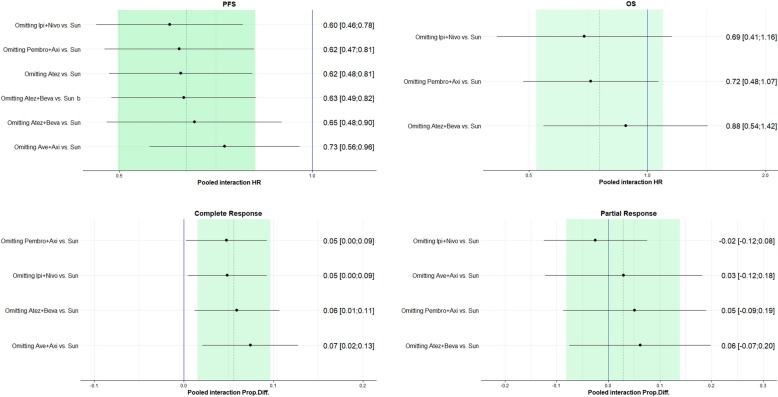
Leave-One-Out-Analyses. Pooled interaction estimates sorted by effect size.

Figure 7 shows the Leave-One-Out-analysis results for HR for death, HR for progression, complete response rate and partial response rate. Pooled interaction estimates were recalculated, with one study omitted each time, and ordered by effect size (low to high). For PFS-HR, pooled interaction estimates slightly changed, with the greatest change obtained by omitting the Avelumab +Axitinib vs Sunitinib study, although all the pooled interaction effects remained significant. As for the HR for death, although the pooled interaction estimates changed by omitting the Atezolizumab+Bevacizumab vs. Sunitinib phase III study, all the pooled interaction effects remained non-significant. As for complete response rate, all the pooled interaction effects remained significant, while as for partial response rate, the pooled interaction estimates changed drastically when omitting the study Ipilimumab+Nivolumab vs. Sunitinib study, but all the estimates remained non-significant.

The results of the network meta-analysis are presented in Table 2. In sRCC, ipilimumab + nivolumab had the highest rank for OS and RC-C, while avelumab + axitinib had the highest rank for PFS and atezolizumab + bevacizumab had the highest rank for PR-R. 

## 5. Discussion

While sunitinib has been the standard of care in the first-line setting of advanced RCC for over a decade [25], with pazopanib representing a non-inferior treatment with a different toxicity profile [26], the therapeutic armamentarium in the first-line treatment setting has expanded to include several novel therapeutic options with evidence of improved efficacy vs. sunitinib, such as cabozantinib [27], axitinib + avelumab [17], axitinib + pembrolizumab [28], ipilimumab + nivolumab [15], atezolizumab + bevacizumab [19]. Indirect comparisons have been published to establish the optimal first-line treatment choice. One meta-analysis [5] including 16 trials (9,343 patients) that were evaluatedfor OS and 25 trials (11,771 patients) that were evaluated for PFS concluded that pembrolizumab plus axitinib (HR: 0.53; 95% CrI: 0.38–0.73), followed by nivolumab plus ipilimumab (HR: 0.63; 95% CrI: 0.50–0.79), had the highest chances of providing the maximum OS benefit vs. sunitinib, while cabozantinib (HR: 0.66; 95% CrI: 0.46–0.94) had the highest chances of providing the maximum PFS advantage vs. sunitinib. Concordant results are presented in the separately published meta-analysis by Hahn et al. [4]. As the number of therapeutic options grows, so does the importance of identifying readily available predictive factors to be potentially included in the therapeutic algorithm for optimal selection of the first-line treatment of RCC patients. While in the ‘targeted’ and ‘cytokine’ eras the therapeutic benefit appeared to be independent on the prognostic risk classification [3], the advent of a novel ‘immunotherapy era’ has shown that treatment efficacy can significantly vary across different risk classes, with ipilimumab + nivolumab being effective in the poor-/intermediate-risk class, but not in the good-risk class [16]. In the sub-group of patients at intermediate-/poor- prognosis, cabozantinib and pembrolizumab plus axitinib were also the most likely to provide the highest treatment benefit in terms of PFS and OS, respectively [5].

PD-L1 expression may also be a valuable predictive factor for anti PD-1/PDL-1 therapy, as shown in another recently published meta-analysis involving 4063 cases RCC patients randomized to anti PD-1/PDL-1 agent vs. sunitinib. OS improvement in unselected cases (HR = 0.65, 95% CI: 0.54–0.79; *p* < 0.001) was lower compared to PD-L1 positive cases (HR = 0.49, 95% CI: 0.36–0.67; *p* < 0.001), although the authors did not perform a formal statistical test to compare these differences. Similarly, pooled analysis of the unselected cases showed a statistically significative improvement in PFS with the use of anti PD1/PDL-1 agents (HR = 0.85; 95% CI: 0.72–0.99), which appeared to be greater in PDL-1 positive patients (HR = 0.65; 95% CI: 0.57–0.74). Response rates also appeared to improve in patients with positive PD-L1 expression (RR = 1.74; 95% CI: 1.21–2.49).

Importantly, sarcomatoid features at histology have been associated with PD-L1 expression, which could provide the biological background supporting our findings [29]. In fact, in a study sample of 26 sarcomatoid RCC specimens, PD-L1 expression was reported in 54% of cases, while among 29 specimens of clear cell RCC without sarcomatoid features, PD-L1 expression was reported only in 17% of cases [9]. Furthermore, sarcomatoid RCC may present a higher mutation burden compared to non sarcomatoid RCC [30], which is a known predictor of immunotherapy efficacy [31].

In our meta-analysis, we provided pooled estimates of outcomes in RCC patients with sarcomatoid features randomized to anti PD-1/PDL-1 agents vs. sunitinib and obtained a pooled PFS-HR of 0.57 and pooled OS-HR of 0.56. Compared to the ITT population, the ratios of pooled PFS-HRs was 0.64 (0.5–0.72), showing a 36% statistically significant improvement in HR-PFS of anti PD-1 agents vs. sunitinib in the sarcomatoid sub-group vs the ITT population. It is important to note that this result is an underestimation of the true ratio of PFS-HR in sarcomatoid patients vs. non sarcomatoid patients as the former are included in the ITT population, and no PFS data in non-sarcomatoid patients were available at the time of our systematic search. No statistically significant interaction was reported for OS-HR between sarcomatoid patients and the ITT population, which requires to be verified using the non-sarcomatoid sub-group rather the ITT population as a comparator for the sarcomatoid sub-group.

The absolute increase in CR rate with anti-PD-1 agent vs. sunitinib was 2.5 times higher in sarcomatoid vs. non-sarcomatoid subgroups (10% vs. 4%), with a statistically meaningful interaction. Achievement of a complete response has an established prognostic value in patients treated with immunotherapy. In a retrospective study including a total of 23 patients treated with high dose interleukin 2 experiencing a complete response and 30 patients showing a partial response, all patients with partial responses, but only four with complete responses had experienced disease recurrence after (>10 years) follow-up. Furthermore, when we tried to rank anti PD-1/PDL-1 agents according to the different efficacy end points considered, ipilumumab + nivolumab ranked the highest in both CR-R and OS-HR.

Our meta-analysis has several types of limitations. First, sub-group data were mostly available in the abstract form and thus have not undergone rigorous peer-review, although grey literature has a recognized importance in systematic reviews [32]. Second, PFS and OS data were unavailable for non-sarcomatoid patients, so we could also estimate ratio between sarcomatoid patients and the entire ITT population. Third, the non-sarcomatoid sub-group also included patients with unknown sarcomatoid status. Fourth, sarcomatoid histology was not centrally reviewed in the trials assessed, nor was it uniformly defined, nor was a stratification factor. Nevertheless, we believe our meta-analysis has the merit to provide pooled estimates of measures of anti PD-1/PDL-1 efficacy in patients with a particularly aggressive histologic variant. As patients at intermediate/ poor prognosis, including those with sarcomatoid features, may also be candidates to cabozantinib, additional data are needed to establish cabozantinib efficacy in patients with sarcomatoid tumors, which are known to express c-MET, one of the biological targets inhibited by cabozantinib [33].

In conclusion, presence of sarcomatoid features is a candidate predictive biomarker for efficacy of anti-PD-1/PDL-1 agents in RCC. Additional research represents a compelling need in order to establish the optimal anti PD-1/PDL-1-based regimen in patients with sarcomatoid RCC, as several combinations are available. Our results suggest that ipilimumab + nivolumab may represent a reference standard for RCTs in sarcomatoid RCC.

## Figures and Tables

**Table 1:** Main characteristics of the trials included. Ipi = Ipilimumab; Nivo = Nivolumab; Sun=Sunitinib; Ave=Avelumab; Axi = Axitinib; Atez = Atezolizumab; Beva = bevacizumab; Pembro = pembrolizumab; Sun = sunitinib.

Ref.	Arms	Number of Patients		Median PFS In Months (Range)		PFS-HR		Median OS In Months (Range)		OS-HR		OR-R%		CR-R%	
		ITT	Sarc	ITT	Sarc	ITT	Sarc	ITT	Sarc	ITT	Sarc	ITT	Sarc	ITT	Sarc
[15,16,24]	Ipi + Nivo	425	60	8,2; (6,9–10)	8,4; (5,2–24)	0,77; (0,65–0,9)	0,38; (0,61–0,97)	35,6	31,2; (23-NE)	0,66; (0,54–0,8)	0,55; (0,33–0,9)	42	56,7	11	18,3
	Sun	422	52	8,3; (7–8,8)	4,9; (4–7)	-	-	26,6; (22,1–33,4)	13,6; (7,7–20,9)	-	-	29	19,2	1	0
[17,22]	Ave + Axi	442	47	13,8; (11,1-NE)	7; (5,3–13,8)	0,69; (0,56–0,84)	0,57; (0,325–1,003)	-	-	0,78; (0,554–1,084)	-	51,4	46,8	3,4	4,3
	Sun	444	61	8,4; (6,9–11,1)	4; (2,7–5,7)	-	-	-	-	-	-	25,7	21,3	1,8	0
[18,23]	Pembro + Axi	432	51	15,1; (12,6–17,7)	-	0,69; (0,57–0,84)	0,29; (0,54–1)	-	-	0,53; (0,38–0,74)	0,58; (0,21–1,59)	59,3	58,8	5,8	11,8
	Sun	429	54	11,1; (8,7–12,5)	8,4	-	-	-	-	-	-	35,7	31,5	1,9	0
[19,21]	Atez + Beva	454	68	11,2; (9,6–13,3)	8,3; (5,4–12,9)	0,83; (0,7–0,97)	0,34; (0,52–0,79)	33,6; (29-NE)	18,3	0,93; (0,76–1,14)	0,56; (0,32–0,96)	37	49	5	10
	Sun	461	74	8,4; (7,5–9,7)	5,3; (3,3–6,7)	-	-	34,9; (27,8-NE)	15; (8,7-NE)	-	-	33	14	2	3
[20]	Atez + Beva	101	15	11,7; (8,4–17,3)	-	0,82; (0,59–1,15)	0,26; (0,63–1,54)	-	-	-	-	-	-	-	-
	Atezo	103	16	6,1; (5,4–13,6)	-	1,18; (0,86–1,63)	0,44; (1–2,3)	-	-	-	-	-	-	-	-
	Sun	101	14	8,4; (7–14)	-	-	-	-	-	-	-	-	-	-	-

**Table 2:** Results of the network meta-analysis. Anti PD-1/PDL-1 agents are ranked from best to worse according to the different efficacy end points considered.

PFS-HR				OS-HR			
Sarcomatoid		ITT		Sarcomatoid		ITT	
Treatments	P-Score	Treatments	P-Score	Treatments	P- Score	Treatments	P- Score
Ave+Axi	0,8678	Ave+Axi	0,8463	Ipi+nivo	0,6825	Pembro+Axi	0,9551
Atez+beva	0,6545	Pembro+Axi	0,8463	Atez+Beva	0,6624	Ipi+nivo	0,708
Pembro+Axi	0,6381	Ipi+nivo	0,6265	Pembro+Axi	0,5981	Atez+Beva	0,2561
Ipi+nivo	0,5404	Atez+beva	0,4719	Sun	0,057	Sun	0,0808
Atez	0,1854	Sun	0,1695				
Sun	0,1139	Atez	0,0395				
CR-R				PR-R			
Sarcomatoid		Non-sarcomatoid		Sarcomatoid		Non-sarcomatoid	
Treatments	P- Score	Treatments	P- Score	Treatments	P- Score	Treatments	P- Score
Ipi+Nivo	0,946	Ipi+Nivo	0,9989	Atez+Beva	0,8438	Atez+Beva	0,9989
Pembro+Axi	0,7159	Pembro+Axi	0,6024	Ave+Axi	0,625	Pembro+Axi	0,6662
Atez+Beva	0,4785	Atez+Beva	0,5292	Ipi+Nivo	0,5621	Ave+Axi	0,5849
Ave+Axi	0,3288	Ave+Axi	0,3209	Pembro+Axi	0,4518	Ipi+Nivo	0,1412
Sun	0,0308	Sun	0,0486	Sun	0,0173	Sun	0,1089
